# Analysis of *C3* Suggests Three Periods of Positive Selection Events and Different Evolutionary Patterns between Fish and Mammals

**DOI:** 10.1371/journal.pone.0037489

**Published:** 2012-05-18

**Authors:** Fanxing Meng, Yuena Sun, Xuezhu Liu, Jianxin Wang, Tianjun Xu, Rixin Wang

**Affiliations:** Laboratory for Marine Living Resources and Molecular Engineering, College of Marine Science, Zhejiang Ocean University, Zhoushan, China; University of Lausanne, Switzerland

## Abstract

**Background:**

The third complement component (C3) is a central protein of the complement system conserved from fish to mammals. It also showed distinct characteristics in different animal groups. Striking features of the fish complement system were unveiled, including prominent levels of extrahepatic expression and isotypic diversity of the complement components. The evidences of the involvement of complement system in the enhancement of B and T cell responses found in mammals indicated that the complement system also serves as a bridge between the innate and adaptive responses. For the reasons mentioned above, it is interesting to explore the evolutionary process of *C3* genes and to investigate whether the huge differences between aquatic and terrestrial environments affected the *C3* evolution between fish and mammals.

**Methodology/Principal Findings:**

Analysis revealed that these two groups of animals had experienced different evolution patterns. The mammalian *C3* genes were under purifying selection pressure while the positive selection pressure was detected in fish *C3* genes. Three periods of positive selection events of *C3* genes were also detected. Two happened on the ancestral lineages to all vertebrates and mammals, respectively, one happened on early period of fish evolutionary history.

**Conclusions/Significance:**

Three periods of positive selection events had happened on *C3* genes during history and the fish and mammals *C3* genes experience different evolutionary patterns for their distinct living environments.

## Introduction

The complement system was first identified as a heat-sensitive factor in fresh serum that ‘complemented’ the effects of specific antibody in the lysis of bacteria and red blood cells. It is a group of humoral and cell surface proteins which play an essential role in innate immune defense against invading microorganisms [1]. In vertebrates, the complement system not only mediates functions contributing to pathogen killing and elimination but also serves as a bridge between the innate and adaptive responses (reviewed in [1], [2], [3], [4]). The vertebrate complement system can be activated through three overlapping pathways: the classical, alternative and lectin pathways [5], [6]. The classical pathway is induced by antigen-antibody interactions, whereas the other two pathways function only in innate immune system. These pathways converge in the formation of the third complement component (C3) convertases, which cleave C3 into the small anaphylatoxin C3a and the large, reactive C3b that may covalently couple to target surfaces [7], [8]. Afterward, the lytic pathway is activated and the membrane-attack complex (MAC) is formed on target cells resulting in cell lysis. And host cells can express both serum and cell surface regulatory proteins to protect against attacking on self cells [9].

C3 is a central protein of the complement system, this versatile and flexible molecule interacts with various proteins to perform its functions. It emerged over 700 million years ago [10] and belongs to the α2-macroglobulin (α2M) family. Members of this family, such as the complement components C3, C4 and C5, the proteinase inhibitor α2M and the insect and nematode thioester-containing proteins (TEPs) [11], are characterized by homologous sequences features, including a unique thioester motif enabling covalent attachment to target particles and a central, highly variable part likely involved in recognition; and, the propensity to undergo conformational changes for distinct protein binding interactions [11], [12], [13]. Researches of crystal structures of human C3 and its derived products revealed thirteen domains. The core of the protein is formed by eight homologous domains, which were named macroglobulin (MG) domains referring to the related immunoglobulin fold and to the family of α2M proteins. The other five domains are crafted onto this core of eight MG domains in two large insertions and one extension [14], [15], [16]. The first insert is located in MG6 and includes the linker region (LNK), the tetra-arginine pro-C3 processing site, the anaphylatoxin (ANA) domain and a linker (α'NT) that connects the ANA domain back to MG6. The second insert is between domains MG7 and MG8 and consists of the CUB (for ‘complement C1r/C1s, Uegf, Bmp1’) domain and the thioester domain (TED) that carries the reactive thioester. The TED domain itself is inserted in loop of the CUB domain [15]. The C345C (for ‘the C-terminal parts of the complement components C3, C4 and C5’) domain at the C-terminal end forms an extension and is connected to MG8 via a short anchor region.

The conformational changes during the conversion of C3 to C3b make many proposed ligands binding sites more accessible. Large conformational changes in several domains are pivotal to the conversion of inactive C3 to active C3b which make C3b more elongated and open than C3. As a direct consequence, putative binding sites for large ligands, such as factor B and properdin, are spatially dispersed, reducing potential steric collisions between them [14]. For the reason of versatility of ligands to C3 and its derived products and lack of structural data, up till now there is not a comprehensive and integrated view of these binding sites.

Since their discovery, complement molecules have been found and studied in a variety of organisms, principally vertebrates. Then the homologs of complement *C3* have been identified from invertebrates including sea squirt, sea urchin, horseshoe crab, coral, and sea anemone [17], [18], [19], [20], [21], [22]. These findings demonstrated that the origin and evolution of the complement system is traced to the earliest radiations of the animal kingdom (reviewed in [23], [24], [25], [26]). These animals differed in immune weapons and lived in distinct environments with huge differences. The innate immune system is the only defense weapon of invertebrates while vertebrates evolved and developed acquired immune system furthermore to against pathogen invasion. As vertebrates, fish faced more intense selection pressures imposed by the aquatic environment in which is filled with countless types and numerous amounts of bacteria and viruses than the terrestrial organism. Moreover, the copy numbers and functions of C3 also varied in different animals. Mammalian C3 is encoded by a single gene, while many teleost fish studied thus far were found to possess multiple forms of C3 which are the products of different genes [27]. Functional studies in trout, carp and seabream showed different binding efficiencies of these C3 isoforms to several complement activating surfaces, suggesting that teleost fish may have evolved a novel strategy to enlarge the innate recognition and destruction of microbes [10], [28]. But pathogenic bacteria and viruses have also evolved countermeasure to evade the immune system. For the importance of the complement system in anti-bacterial defense, many of these evasion pathogens are directed against complement components either by blocking the central activation step of C3 to C3b or indirectly blocking C3 convertase by attracting host convertase regulators to their surfaces [29]. Therefore, the study on the evolutionary history of *C3* would contribute to further understanding the evolution of innate immune system in both invertebrates and vertebrates. We also explored whether the evolutionary pattern of *C3* between fish and mammals are different for their distinct living environments. And duplicated genes are important in supplying raw genetic material to biological evolution [30]. Thus, it is interesting to explore the evolution patterns of duplicated fish *C3* gene copies.

## Results

### Evolutionary analyses revealed three periods of positive selection events on C3 genes

**Figure 1:**
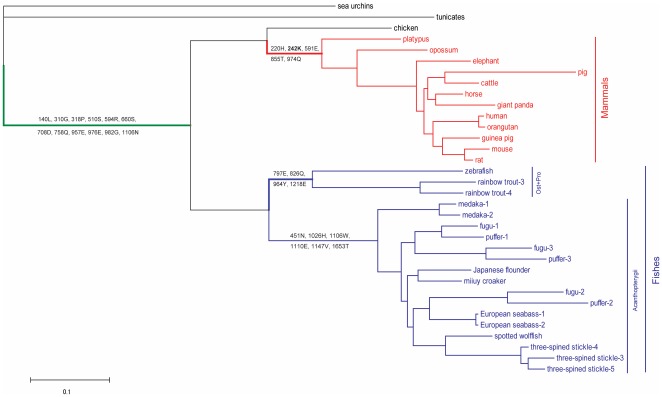
The putative gene tree for *C3* reconstructed by Bayesian approach with no constraints on the topology. The Darwin selection pressures were detected by the branch-site models in the ancestral lineages to vertebrates (in green), mammals (in red) and ostariophysian together with protacanthopterygian (Ost+Pro) fishes (in blue). The positive selected sites with posterior probabilities larger than 0.95 (PP>0.99 in bold) were showed on the corresponding lineages. The synonymous substitution (*d*_N_), non-synonymous substitution (*d*_S_) of nucleotides and the ratio of *d*_N/_ *d*_S_ of these ancestral lineages were showed. The sequences of mammalian (red) and fish *C3* (blue) were then tested by site-model tests in next analysis, respectively.

The Bayesian tree reconstructed with the thirty-four *C3* sequences from twenty-five different species is well supported with high posterior probabilities (PP = 1.00) in all nodes except one node (PP = 0.98). And the reconstructed mammalian phylogeny part is in accordance with the established mammalian species tree [31] and the fish phylogeny part can be divided into two parts, the actinopterygian fish which is the most dominant class of fish in term of species number and the ostariophysian fish which is second-largest super-order of fish together with its relative, the protacanthopterygian fish. We thus used this established Bayesian tree in all subsequent analyses. To explore the possibility of different selection on *C3* in fish and mammals for their huge differences of living environments, we estimated the ratio (*ω*) of nonsynonymous to synonymous substitution rates by a likelihood method. Firstly, under the one-ratio model assuming of a uniform *ω* for all branches of the tree, the value of *ω* was estimated to be 0.250, which is significantly smaller than 1 (data not shown). This result suggests that an overall strong purifying selection exists in *C3* genes. Secondly, a free-ratio model that allows different *ω* values for each lineage among the tree fits the data significantly better than the simpler one-ratio model. In this analysis, extreme high *ω* values, strong signal of positive selection, were detected among three ancestral lineages leading to all vertebrates, mammals and ostariophysian together with protacanthopterygian fishes, respectively (data not shown). Thirdly, the branch-site model was conducted to detect positive selection that affects only a few sites along our interesting lineages mentioned above (namely the foreground branches). For all of these three ancestral lineages, the branch-site models in which allows positive selection on the foreground lineages (see Fig. 1) all showed significantly better fit than their corresponding branch-site null models which do not allow such positive selection. The Bayes empirical Bayes (BEB) approach was used to calculate the posterior probabilities (PP) that each site belongs to the site class of positive selection on the foreground lineages. Totally, twenty-one positively selected sites were detected among these three ancestral lineages (model 2, 4 and 6 in Table 1). To test whether different codon frequency models, which allow different codon frequencies and/or base bias on different codon position, have effect on the results, new branch-site tests under different codon frequencies using the *CodonFreq* parameter were conducted. The results proved the correctness of previous analysis (data not shown). The detected positively selection sites strongly indicated the presence of three periods of positive selection on *C3* during animal evolutionary process.

**Table 1:** Likelihood ratio tests of branch-site models on *C3* genes.

Model	np	ln*L*	Model comparison	2Δ(ln*L*)	*P*-value	positive selected sites
1: Null-vert	69	−79225.31				
2: Vert	70	−79210.21	1 and 2	30.20	**1.0E-6**	140L, 310G, 318P, 510S, 594R, 660S, 708D, 758Q, 957E, 976E, 982G, 1106N
3: Null-Mam	69	−79231.38				
4: Mam	70	−79211.96	3 and 4	38.84	**0.0**	220H, **242K** ^5^, 591E, 855T, 974Q
5:Null-OP	69	−79236.76				
6: OP	70	−79209.22	5 and 6	55.08	**0.0**	797E, 826Q, 964Y, 1218E,
7:Null-Acan	69	−79241.15				
8:Acan	70	−79216.70	7 and 8	48.90	**0.0**	451N, 1026H, 1106W, 1110E, 1147V, 1653T
9: Null-Seabass2	69	−79241.51				
10: Seabass2	70	−79230.22	9and 10	64.58	**0.0**	n/a
11: Null-Stickle5	69	−79234.35				
12: Stickle5	70	−79230.63	11 and 12	7.44	**0.0063**	n/a

Note: np number of parameters, ln*L* ln[likelihood] value, 2Δ(ln*L*) twice the difference of ln[likelihood] between the two models compared, vert, mam, OP, and Acan the ancestor branches of the vertebrates, mammals, ostariophysian together with protacanthopterygian fishes, and acanthopterygians fishes, respectively, examined in present study, seabass2 the seabass *C3-2*, stickleback5 the three-spined stickleback *C3-5*.The *P*-values<0.01 are shown in boldface. The human *C3* sequence was used as reference to mark the positions of the positive sites in all cases. Sites with the *P*-values<0.05 are shown and those with *P*-values<0.01 are in boldface.

### No evidence of positive selection was detected on duplicated fish C3 genes

To explore the possible positive selected sites on duplicated fish *C3* genes, a free-ratio model was first used to evaluate the *ω* values of all lineages. Only the lineages of European seabass *C3-2* and three-spined stickleback *C3-5* showed *ω* values larger than 1 (1.15 and 1.14, respectively). Then the branch-site models were conducted to detect whether positive selected sites existed in these two lineages. Although the branch-site models were not rejected (*P*<0.01, Table 1), no sites were detected with PP values larger than 0.95.

### Site-models reveal different selection patterns on C3 between fish and mammals

**Figure 2:**
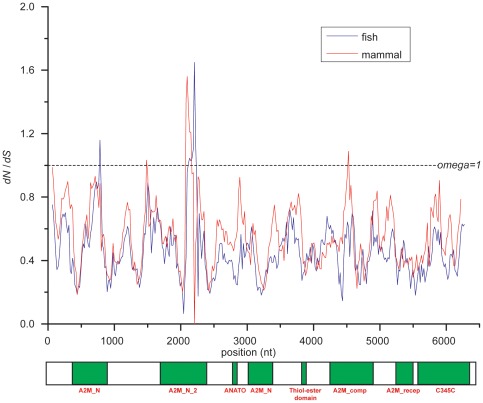
Sliding window analysis of variation in omega value along fish and mammalian *C3* genes. It was set to be 90 bp for the window size and 36 bp for the step size. Beneath the plot is a schematic of the *C3* gene, which illustrates the distribution of the characteristic domains.

By the sliding window method, we separately calculated the values of *ω* among fish and mammalian *C3* codons (Fig. 2). The estimates of *ω* values were found to be larger than one at the A2M_N_2 domain for both of mammals and fish, also at A2M_N domain for fish and A2M_comp domain for mammals, indicative of elevated evolutionary rates at these domains.

To explore whether the different living environments act on the evolutionary progress of *C3* in jawed vertebrates or not, the site models were used to detect the possible positive selection in fish and mammals, respectively. The site models treat the *ω* ratio for any site (codon) in the gene as a random variable from a statistical distribution, thus allowing *ω* to vary among codons [32], [33]. Positive selection is defined as presence of some codons at which *ω*>1 (in model M2a or M8). An LRT is constructed to compare a null model that does not allow for any codon with *ω*>1 (M1 or M7) against a more general model that does. Site models were conducted on subset of mammal or fish *C3* sequences, respectively.

For mammals, no positive selected sites were detected by either M2a model or M8 model (Table 2, mammal subset). However the fish *C3* showed different evolutionary pattern. The LRT test statistic (2Δln*L*) of M7-M8 comparison of fish subset was 150.34 (*P*<0.01, Table 2, fish subset), indicating that positive selected sites probably existed in fish. Then the BEB approach detected seven sites under positive selection on fish *C3* based on model M8, of which six had PP values >0.95 (293I, 294T, 432G, 433P, 737T, 802D, and 1360S, see Table 2, model M8 of fish subset). These results provide evidence of different selection pressures on fish and mammal *C3*, indicating the strong Darwin selection pressures on fish *C3* genes.

**Table 2:** Site model tests on subset of fish or mammalian *C3* genes.

Model	np	ln*L*	parameter	Model comparison	2Δ(ln*L*)	*P*-value	positive selected sites^1^
**Data set: mammals**							
M0 (one ratio)	27	−41738.40	*ω* = 0.178				None
M1a (nearly neutral)	28	−41035.20	*p*_0_ = 0.794, (*p*_1_ = 0.206)				
M2a (positive selection)	30	−41035.20	*p*_0_ = 0.794, *p*_1_ = 0.000, (*p*_2_ = 0.206), *ω*_2_ = 1.00	M2a and M1a	0.0	1.0	Not allowed^2^
M3 (discrete)	31	−40858.91	*p*_0_ = 0.266, *p*_1_ = 0.530, (*p*_2_ = 0.204), *ω*_0_ = 0.027, *ω*_1_ = 0.132, *ω*_2_ = 0.706	M3 and M0	1758.98	**0.0**	
M7 (beta)	28	−40929.27	*p* = 0.706, *q* = 2.565				
M8 (beta & w>1)	30	−40851.91	*p_0_* = 0.890, *p* = 1.182, *q* = 7.291, (*p_1_* = 0.110), *ω* = 1.000	M7 and M8	154.72	**0.0**	None
**Data set: fish**							
M0 (one ratio)	41	−54906.39	*ω* = 0.309				None
M1a (nearly neutral)	42	−53795.95	*p*_0_ = 0.683, (*p*_1_ = 0.317)				
M2a (positive selection)	44	−53795.95	*p*_0_ = 0.683, *p*_1_ = 0.265, (*p*_2_ = 0.052), *ω*_2_ = 1.00	M2a and M1a	0.0	1.0	Not allowed
M3 (discrete)	45	−53584.70	*p*_0_ = 0.320, *p*_1_ = 0.455, (*p*_2_ = 0.225), ω_0_ = 0.058, *ω*_1_ = 0.273, *ω*_2_ = 0.987	M3 and M0	2643.38	**0.0**	
M7 (beta)	42	−53637.19	*p* = 0.694, *q* = 1.342				
M8 (beta & w>1)	44	−53562.02	*p_0_* = 0.864, *p* = 1.045, *q* = 3.305, (*p_1_* = 0.136), *ω* = 1.197	M7 and M8	150.34	**0.0**	293I, 294T, 432G, 433P, 737T, **802D**, 1360S

Note: the site models were conducted on mammalian or fish *C3* gene sequences with two invertebrate *C3* as out-group, respectively.

1 : only the sites with PP>0.95 were shown and those with PP>0.99 are in bold.

2 : this means that the models which allowed the positively selected sites exist in sequences did not pass the likelihood ratio test and thus the positively selected sites were not allowed to exist.

## Discussion

In this study, we surveyed the thirty-four *C3* sequences of twenty-five different species to explore the evolutionary process of *C3* genes and to examine whether the different environments had caused different selection pressures between aquatic and terrestrial organisms. Because the aquatic environments contain countless kinds of bacteria and virus and fishes are armed with less developed adaptive immune system comparing with mammals, one may expect the innate immunity including the complement system of fish plays much more important roles in defense against pathogen invading. The site-models tested on fish and mammalian *C3* genes revealed that these two groups of vertebrates which are flourishing in the aquatic and terrestrial environments, respectively, experienced different evolutionary patterns. No evidence of positive selection was detected in mammalian *C3* while seven sites were found to be under positive selection in fish *C3* (Table 2), indicating the different evolutionary pressure on these two groups whose living environments differed hugely.

Molecular evolution analyses were also conduced to explore the possible evolutionary process of *C3*. Many positively selected sites were detected among the common ancestral lineages to the vertebrates, mammals and protacanthopterygian and ostariophysian fishes, indicating that episodic positive selection events had happened during the *C3* evolution along these lineages. The first period of positive selection happened with the emergence of vertebrates. From the evolutionary standpoint, the complement system is present in both of vertebrates and a wide range of invertebrates. Unlike the vertebrates, the complement system of invertebrate was more primitive although they showed some complexity and diversity [34], [35]. Those invertebrate complement systems lack the antibody and thus the classical pathway, which is based on the antibody-recognizing activation cascade, and seem to represent a prototypic opsonin system composed of C3 and its activation cascades that seem to correspond to mammalian lectin and/or alternative pathways [36], [37]. The ancient origin of *C3* gene can be traced back to cnidarians, one of the most primitive metazoan members [18], [38] and it has been evolutionarily retained in both deuterostomes and some lineages of protostome, such as arthropods (horseshoe crab) [22] and mollusks [39], [40]. The antimicrobial activities of the invertebrate C3, through a complement-mediated phagocytosis, have been proven only in the sea urchin (echinoderm) [36] and ascidians (urochordate) [20]. And no evidence of direct cytolytic activity has been proven in invertebrate primitive complement system. Thus, we speculate that with the evolvement of antibody in the vertebrate, the complement system had experienced the first period of positive selection on the ancestral vertebrates to evolve the classical pathway of C3-activation and the cytolytic pathway. These huge advances of immunity, emergence of antibody (adaptive immunity) and the classical pathway of activating complement system (innate immunity), promoted the flourish of the ancestral vertebrates.

The second period of positive selection happened on the early period of fish evolutionary history. The ancestral lineage leading to ostariophysian and protacanthopterygian fish also showed positively selected sites, indicating one more period of positive selection event on *C3*. Besides that, six positive selection sites were also detected among the ancestral lineage leading to all actinopterygian fish (Table 1). The positive selection sites detected among the ancestral lineages to fish reflected the second period of positive selection on the early period of fish *C3* evolutionary history. Fish are most primitive groups of jawed vertebrates, their complement system had evolved all three C3-activation pathways and the cytolytic pathway, showing many of the effecter activities recognized in the mammalian complements, such as target cell killing, opsonization, and anaphylatoxic leukocyte stimulation [41]. Although fish complement system has showed a high degree of structural and functional conservation of the complement pathways and their components comparing with mammals, striking features of the fish complement system were also unveiled, including prominent levels of extrahepatic expression and isotypic diversity of the complement components. Whole-genome duplication (WGD) is to be one of the major evolutionary events that shaped the genome organization of vertebrates. Three WGD events have been proposed in ancient vertebrate history: two at the origin of the group and a third specific to fish [42]. The distinctiveness of fish complement system probably was the long evolution results of the ancient fish-specific genome duplication (FSGD) under the aquatic environment. Studies of the genomes of zebrafish and two close relative Tetraodontiformes (*Tetraodon* and *Takifugu*) [43], [44] have confirmed that ray-finned fish underwent a FSGD some 320–400 million years ago which might explain their evolutionary success.

The third period of positive selection of *C3* happened on ancestral mammalian lineage. After its discovery, intensive and detailed researches have focused on human complement system, more than 30 plasma and cell-surface complement proteins have been found. The complement system is known to be a highly sophisticated host-defense system that is engaged in both the innate and adaptive immunities [45], [46]. It involves in a range of functions from direct cell lysis to the enhancement of B and T cell responses [47], [48]. Given the multiple pathways of activation and the versatile functions of derived products of complement members, regulation of the complement system is complex and necessary. Activation of complement is critical for protection against pathogen infection; however, inappropriate activation of complement contributes to the pathogenesis of immunological and inflammatory diseases [49]. To limit host destruction, the system makes use of both serum and cell surface regulatory proteins. The cell-expressed members of this family are membrane cofactor protein (MCP; CD46), decay accelerating factor (DAF; CD55) and complement receptors one (CR1; CD35) and two (CR2; CD21) while the plasma members are C4-binding protein (C4BP) and factor H (FH). Complement regulators at the C3 cleavage step possess cofactor activity (CA) or decay accelerating activity (DAA). Almost all mammalian cells express regulators of complement to protect against attacking on self [9]. Nearly half of the complement proteins participate in regulation. And five positive selection sites were detected in ancestral mammalian lineage (Fig. 1). The presumed positively selected residue 1193 is on the surface of TED domain and located in the proposed binding site (residues 1187–1249) for factor H [50]. Other four positive selection sites were located in the internal of MG4, MG7 and CUB domains, respectively. Factor H, decay-accelerating factor (DAF, CD55) and complement receptor 1 (CR1, CD35) are three important members of regulators of complement activity which inhibit the C3 convertase activity [51]. Different from the diversity of C3 and quantitatively evolution manner in fish, mammals took more elaborately regulation on the existing members of complement system.

In conclusion, although *C3* gene is conserved from invertebrate to vertebrate, it had happened three periods of positive selection events during animal evolutionary history. Two happened on the ancestral lineages to all vertebrates and mammals, respectively, one happened on early period of fish evolutionary history. For the reason of huge differences between aquatic and terrestrial environments, the *C3* genes of fish and mammals had experienced different evolution patterns.

## Materials and Methods

### Ethics statement

All work was conducted with the approval of the Animal Ethics Committee.

### Taxonomic Coverage

We screened and obtained partial length of *C3* from the spleen cDNA library of miiuy croaker, *Miichthys miiuy* [52]. The 5′ and 3′ RACE-PCR was performed following the manufacturer's instructions to obtain the full length of miiuy croaker *C3* (with accession number JQ033711). Furthermore, we retrieved eighteen *C3* sequences from nine fish species and twelve sequences from twelve mammals, together with three *C3* from two invertebrates and one bird, from GenBank (http://www.ncbi.nlm.nih.gov/Genbank/) or Ensembl (http://www.ensembl.org/) database for evolutionary analyses (see Table 3).

**Table 3:** Taxonomy of species and accession numbers of *C3* sequences used in this study.

Taxonomy	Common name	Species name	Accession Number
Class Echinoidea			
Order Echinoida	purple sea urchins	*Strongylocentrotus purpuratus*	NM_214521.1
Class Ascidiacea			
Order Enterogona	ascidian	*Ciona intestinalis*	NM_001032512.1
Class Aves			
Order Galliformes	chicken	*Gallus gallus*	NM_205405.1
Class Mammalia			
Order Rodentia	mouse	*Mus musculus*	ENSMUST00000024988
	rat	*Rattus norvegicus*	NM_016994.2
	guinea pig	*Cavia porcellus*	NM_001172903.1
Order Didelphimorphia	opossum	*Monodelphis domestica*	ENSMODT00000034216
Order Perissodactyla	horse	*Equus caballus*	ENSECAT00000007684
Order Cetartiodactyla	cattle	*Bos taurus*	ENSBTAT00000022979
	pig	*Sus scrofa*	ENSSSCT00000014800
Order Carnivora	giant panda	*Ailuropoda melanoleuca*	ENSAMET00000007996
Order Proboscidea	elephant	*Loxodonta africana*	ENSLAFT00000010468
Order Monotremata	platypus	*Ornithorhynchus anatinus*	ENSOANT00000009742
Order Primates	human	*Homo sapiens*	ENST00000245907
	orangutan	*Pongo pygmaeus*	ENSPPYT00000011025
Class Actinopterygii			
Superorder Acanthopterygii			
Order Tetraodontiformes	spotted green pufferfish	*Tetraodon nigroviridis*	ENSTNIT00000017333
			ENSTNIT00000017266
			ENSTNIT00000021460
	tiger puffer	*Takifugu rubripes*	ENSTRUT00000027127
			ENSTRUT00000004988
			ENSTRUT00000045315
Order Pleuronectiformes	Japanese flounder	*Paralichthys olivaceus*	AB021653.1
Order Perciformes	miiuy croaker	*Miichthys miiuy*	JQ033711
	spotted wolffish	*Anarhichas minor*	AJ30957.1
	European seabass	*Dicentrarchus labrax*	HM563078.1
			HM563079.1
Order Gasterosteiformes	three-spined stickleback	*Gasterosteus aculeatus*	ENSGACT00000024968
			ENSGACT00000024978
			ENSGACT00000024823
Order Beloniformes	medaka	*Oryzias latipes*	NM_001105082.1
			NM_001105083.1
Superorder Ostariophysi			
Order Cypriniformes	zebrafish	*Danio rerio*	NM_001037236.1
Superorder Protacanthopterygii			
Order Salmoniformes	rainbow trout	*Oncorhynchus mykiss*	AF271080.1
			U61753.2

### Evolutionary Analysis

The miiuy croaker *C3* sequence with those of other species retrieved from public database was aligned under codon model with MUSCLE software for its high accuracy and speed [53], [54]. With the alignment, the phylogenetic tree was reconstructed by using MrBayes3.1 [55]. For Bayesian inference, we evaluated the best-fit model as GTR+I+Γ by Bayesian information criterion (BIC) using jModeltest [56], [57]. The MrBayes3.1 program was run with 5,000,000 generations with a burn-in of 25%. Ancestral sequences were reconstructed using the Bayesian method [58] implemented in the BASEML program in PAML 4.1 [59]. To investigate the evolutionary process of *C3* and whether the different environments had caused different selection pressures between aquatic and terrestrial organisms, such as teleost and mammals, we employed the codon-based method to estimate the ratio of nonsynonymous and synonymous substitutions (*ω*) using PAML4 [59]. The subset of mammalian and fish *C3* sequences, both of which used the two invertebrates *C3* as out-group, were analyzed by site-models to detect the possible selective pressures on these two groups of animals with huge difference in living environments. Basically, a free-ratio model was first employed to allow the *ω* ratios to vary for each branch. Then, the likelihood ratio test (LRT) was used to evaluate whether this model fits the data significantly better than the one-ratio model which assumes all branches have only one ratio. Then the branch-site model was used to detect positive selection that affects the interesting foreground lineages (for example, the ancestor lineages to teleost or mammals or the duplicated *C3* lineages of some fish). Finally, six site models were applied to subset of teleost and mammalian *C3* sequences, respectively, to examine the possible positively selected sites among those lineages. In all cases, twice the difference of log-likelihood values (2Δln*L*) between the two models was calculated following a chi-squared distribution with degrees of freedom equaling the difference in parameter numbers estimated in the nested models.

### Sliding windows analysis

To explore further the heterogeneous selection pressure across codons of *C3* genes between fish and mammals, a sliding window analysis of *ω* values was conducted using the Nei and Gojobori method [60]. Sliding windows were implemented in the program SWAAP 1.0.2 [61] with window and step sizes of 90 and 36 nucleotides, respectively.
